# Influence of occlusal reduction on pain after endodontic treatment: a systematic review and meta-analysis

**DOI:** 10.1038/s41598-021-93119-6

**Published:** 2021-07-07

**Authors:** Nayane Chagas Carvalho Alves, Sirley Raiane Mamede Veloso, Silmara de Andrade Silva, Andressa Cartaxo de Almeida, Christianne Tavares Velozo Telles, Kaline Romeiro, Gabriela Queiroz de Melo Monteiro, Diana Santana de Albuquerque

**Affiliations:** Faculdade de Odontologia de Pernambuco, Universidade de Pernambuco, Av. Prof. Luís Freire, 700-Cidade Universitária, Recife, PE 50740-540 Brazil

**Keywords:** Dentistry, Endodontics

## Abstract

The purpose of this systematic review was to analyze the influence of occlusal reduction on the postoperative pain levels after endodontic treatment (instrumentation and obturation of the root canal system). This review followed the PRISMA statement and was registered at PROSPERO (CRD42018107918). Two independent reviewers searched the Lilacs, Cochrane Library, PubMed (Medline), Web of Science, Scopus, Scielo, and ScienceDirect for articles published until April 2021. The research question was, "Does occlusal reduction decrease postoperative pain in endodontically treated teeth?". Only randomized clinical trials were included. The RevMan 5 program was used for meta-analysis, calculating the relative risk (RR) and 95% confidence interval (CI) of the dichotomous outcome (presence or absence of pain). The search strategies retrieved 4114 studies. Twelve studies were included for qualitative analysis and nine for quantitative analysis. The meta-analysis results did not reveal a significant difference in the reduction of postoperative pain levels for endodontic instrumentation at 6, 12, 24, 48 h and for endodontic obturation at 6 or 12 h after occlusal reduction. According to the GRADE tool, the analyzed outcome was classified as having a moderate level of certainty. It is concluded that occlusal reduction does not interfere with postoperative pain levels after endodontic treatment.

## Introduction

Postoperative pain results from an acute inflammatory response in periradicular tissues^1^ and is considered a complication of endodontic procedures with a reported incidence ranging from 3 to 58%^2^. The causes of postoperative pain include inflammation in the periapical tissues^3^, perturbations in the endodontic microbiota, and chemical or mechanical injury caused by intracanal procedures^1^.

Another possible cause of pain after endodontic treatment is excessive occlusal forces in the tooth, which can trigger mechanical allodynia and extended postoperative pain^4,5^. Allodynia is defined as a reduction in pain threshold in response to innocuous mechanical or thermal stimulation. Preoperative mechanical allodynia in patients presenting irreversible pulpitis has been reported around 57%^4^. Periradicular mechanical allodynia can contribute to the early stages of odontogenic pain because of the inflammation of vital pulpal tissue. Furthermore, a large amount of dental tissue is removed during endodontic treatment to access the root canal system, which can cause significant changes in the occlusal status^6^.

Hyperocclusion/occlusal trauma is a potential source of pain and fracture^7^. Previous studies have analyzed pain after endodontic treatment followed by an occlusal reduction (removal of all occlusal contacts) or occlusal adjustment (maintenance of normal occlusal contacts). The goal was to decrease the intensity of occlusal forces on the endodontically treated tooth and reduce the incidence of postoperative pain^5,8–16^. However, there is no consensus on the need for an occlusal reduction after endodontic treatment^5,8,11–13,16^.

The presence of persistent postoperative pain increased the burden on the patients who experience it and is associated with more significant healthcare^17^. Thus, the purpose of this systematic review was to analyze the influence of occlusal reduction on postoperative pain levels after endodontic treatment. The following hypotheses were raised: (1) occlusal reduction interferes with pain after endodontic instrumentation; (2) occlusal reduction interferes with pain after obturation of the root canal system.

## Materials and methods

### Protocol and registration

This review was performed following the recommendations of the Cochrane Collaboration for systematic reviews^18^, and it was reported according to the Preferred Reporting Items for Systematic Reviews and Meta-Analyses (PRISMA) statement^19^. The study was registered in the PROSPERO (International Prospective Register of Systematic Reviews) database under the registration number CRD42018107918.

### Eligibility criteria

The research question of this study was “Does occlusal reduction decrease postoperative pain in endodontically treated teeth?” and the PICOS of the study was then established. The population consisted of patients with teeth submitted to endodontic treatment. The intervention was an occlusal reduction (removal of all occlusal contacts) compared to occlusal adjustment (maintenance of occlusal contacts). The evaluated outcome was postoperative pain, and only clinical trials were included in the study design.

The inclusion criteria were: (1) only randomized clinical trials; (2) studies that compared occlusal reduction after endodontic treatment with a control group occlusal adjustment; (3) studies that evaluated postoperative pain. The exclusion criteria were as follows: (1) case report and series; (2) abstracts; (3) review articles; (4) in vitro studies; (5) discussions; (6) interviews; (7) editorials or opinions, and (8) clinical trials that involved patients who reported bruxism or clenching, patients treated with antibiotics or analgesics over the past 24 h, teeth associated with swelling, presence of periodontal disease or mobility grade 1, and treatment with technical problems (e.g., root canal transportation, ledging, perforation, zipping, file fracture).

### Information sources and search strategy

The databases searched were Lilacs (Latin American and Caribbean Health Sciences Literature database), Cochrane Library, PubMed (Medline), Web of Science, Scopus, Scielo, and ScienceDirect. Additionally, the reference list of the included studies was checked to identify possible relevant studies. The records were identified on the databases within a five-day interval, and the date of the last search was April 26, 2021. No software was used to retrieve searches, and manual searches were done within each database.

The search strategy was defined by performing a preliminary search using specific keywords for occlusal reduction or adjustment. However, this strategy did not retrieve relevant studies. Thus, a search was performed based on terms related to endodontic treatment and postoperative pain, in general, using "Text Words" and "Mesh Terms". The search strategies used for each database are described in Table 1.

**Table 1:** Electronic databases used and search strategy.

Database	Search strategy
LILACS	((tw:(endodontology)) OR (tw:(endodontics)))) AND (tw:(Postoperative Pain)) OR (tw:(Postoperative Pains))
Cochrane Library	((((postoperative pains) OR postoperative pain) OR postoperative pain)) AND ((endodontics) OR Endodontology)
PubMed (Medline)	((((endodontics[MeSH Terms]) OR endodontology[Title/Abstract]) OR endodontics[Title/Abstract])) AND (((postoperative pain[MeSH Terms]) OR Postoperative Pain[Title/Abstract]) OR Postoperative Pains[Title/Abstract])
Web of Science	TI = ("Postoperative Pain" OR "Postoperative Pains") AND TS = (Endodontics OR Endodontology)
Scopus	TITLE-ABS-KEY((“Postoperative Pain”) OR (“Postoperative Pain”)) AND TITLE-ABS-KEY((Endodontics) OR (Endodontology))
Scielo	(ti:(“Dor Pós-Operatória” OR “Dolor Posoperatorio” OR “Pain, Postoperative”)) AND (ti:((Endodontia OR Endodoncia OR Endodontics))
ScienceDirect	Title, abstract or keywords((((postoperative pains) OR postoperative pain) OR postoperative pain[MeSH Terms])) AND ((endodontics) OR Endodontology)

The electronic searches were performed until April 2021 with no restrictions of the start date. Studies published in English, Spanish, and Portuguese were included.

*MeSH* medical subject heading.

### Study selection and data collection

Two independent researchers (N.C.C.A. and A.C.A.) performed the electronic search and selected studies based on titles and abstracts that answered the research question. The duplicate removal was performed using online software (Rayyan—https://www.rayyan.ai/) before the records were screened. After the initial search, the relevant data were extracted. All initial steps were performed independently. The following data were collected: (1) initial diagnosis; (2) type of tooth evaluated; (3) technique of chemical–mechanical preparation; (4) obturation techniques; (5) restorations; (6) intervention/comparison; (7) method of postoperative pain assessment; (8) moment of postoperative pain assessment (post obturation and post instrumentation); (9) presence of pain. Data on pain was extracted, regardless of the scale used and the type of variable (quantitative or qualitative). In case of missing information in the included articles, e-mails were sent to the corresponding authors. Contact was waited up to 15 days.

### Risk of bias in individual studies

The methodological quality was examined independently by two reviewers (N.C.C.A. and A.C.A.) using the Cochrane Collaboration's tool for assessing the risk of bias. Discrepancies were resolved by a third reviewer (S.R.M.V.). In this tool, the aspects of bias risk are evaluated individually without assigning scores. They are divided into seven domains: random sequence generation, allocation concealment, blinding of participants and personnel, blinding of the outcome assessment, incomplete outcome data, selective reporting, and other sources of bias. Each domain was classified as having a low, unclear, or high risk of bias.

### Summary measures

The extracted data were analyzed using the Review Manager (RevMan) 5.3 software (The Cochrane Collaboration, Copenhagen, Denmark). The relative risk (RR) and 95% confidence interval (CI) were calculated for each study. The data of the eligible studies were dichotomized as the presence or absence of postoperative pain.

The I^2^ statistic was used to evaluate the percent variation among studies due to heterogeneity, with 0–40% corresponding to might not be important heterogeneity, 30–60% may represent moderate heterogeneity, 50–90% may represent substantial heterogeneity, and 75–100% considerable heterogeneity^18^.

A sensitivity analysis was done to identify the sources of heterogeneity. However, even after removing clinical and methodological differences (Instrumentation protocol, Stage of endodontic treatment, Type of scale) from the studies from Raza et al.^23^; Parirokh et al.^5^; Emara et al.^11^, no significant differences were observed in the results.

### The certainty of evidence assessment

According to the Grading of Recommendations, Assessment, Development, and Evaluation (GRADE) approach^20^, the strength of evidence was evaluated. The summary of the findings (SoF) table was constructed with the software GRADEpro GDT: GRADEpro Guideline Development Tool; McMaster University, 2015 (developed by Evidence Prime, Inc.). Each GRADE criterion was assessed individually and then computed for the certainty of the evidence. The GRADE approach classifies the certainty of evidence in one of the following four grades: high, moderate, low, or very low to achieve transparency and simplicity.

## Results

### Study selection

The initial search of the databases retrieved 4114 articles. After the removal of duplicates, 2987 articles remained. Titles and abstracts were read, and 13 articles were potentially eligible at this stage. After reading the full text, one article was excluded because of language (Persian)^14^. Finally, 12 articles^5,9–11,13,15,16,21–24^ were considered eligible for data extraction.

The number of patients that experienced postoperative pain varied for endodontic instrumentation. After 6 h, the number of patients with pain varied from 15 to 115, 13–87 after 12 h, 3–72 after 24 h, and 6–54 patients after 48 h. For endodontic obturation, the number of patients with pain after 6 h varied from 8 to 54 and 4–10 after 12 h. Doubts arose in two studies^11,15^, and the authors were contacted. Only one^11^ provided the requested information.

**Figure 1:**
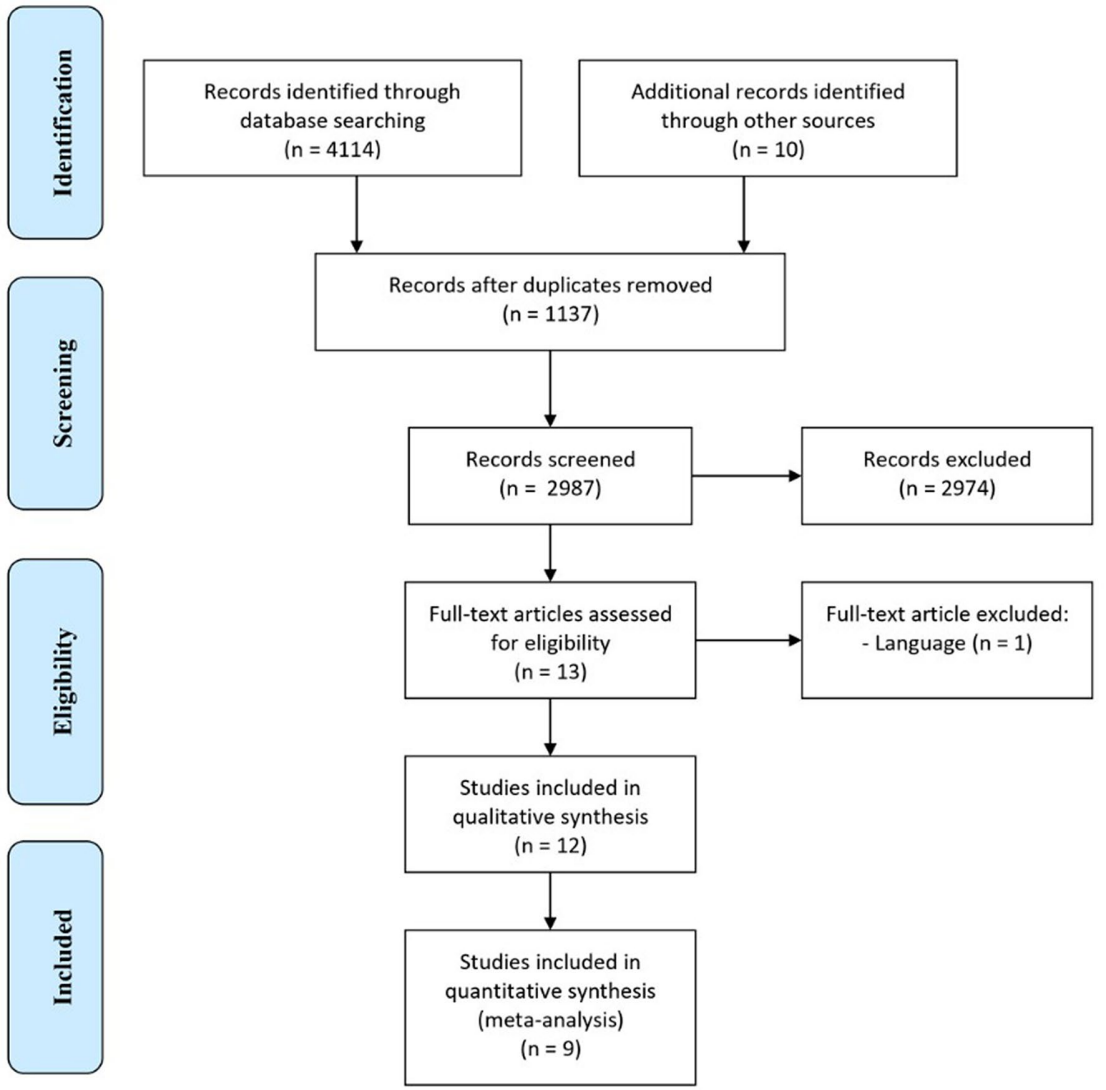
Preferred reporting items for systematic reviews and meta-analyses flow diagram.

Nine studies were included for quantitative synthesis^5,9–11,15,16,21,23,24^. The PRISMA flow diagram showing the complete selection process and inclusion of the articles is illustrated in Fig. 1.

### Characteristics of the included studies

The 12 studies selected were analyzed qualitatively (Table 2). A total of 1461 endodontic treatments were performed in 1461 patients. However, this number does not comprise the data from Rosenberg et al.^13^ because they did not report the number of teeth submitted to endodontic treatment in the experimental groups. Sample size calculation was not reported in five studies^9,10,13,16,23^ and four studies^9,10,13,16^ did not mention ethical considerations.

**Table 2:** Evidence table summarizing the characteristics of the included studies.

Study	Diagnosis	Teeth	Instrumentation protocol			Restorative material	Postoperative pain assessment			Groups (n)		Results
			Preparation	NaOCl concentration (%)	Intracanal medication (ICM)		Stage of endodontic treatment	Type of scale	Experimental period	Control group (occlusal adjustment)	Study group (occlusal reduction)	
Ahmed et al. (2020)^21^	Symptomatic irreversible pulpitis with sensitivity to percussion	Mandibular posterior teeth	Crown-down technique Manual files and Revo-S rotary system	2.5	No ICM (sterile cotton pellet)	After instrumentation: MD-Temp (based on zinc oxide)	After instrumentation	Numerical rating scale	After instrumentation: 6, 12, 24 and 48 h	154 (using articulating paper)	154 (not report the extent of occlusal reduction)	Significant results of reduction in postoperative pain levels 12 and 24 h after instrumentation
						After obturation: Final coronal restoration. Not reported	After obturation		After obturation: 6 and 12 h			
Arslan et al.^8^	Symptomatic apical periodontitis	Molars	ProTaper Universal and K files in some cases	1.25	–	Fluid resin and nanohybrid resin	After obturation	Visual analog scale	1st, 3rd, 5th and 7th day	11 (using a computerized analysis system)	13 (not report the extent of occlusal reduction)	No statistical significance
Asghar et al. (2014)^9^	Symptomatic irreversible pulpitis	Posterior maxillary and mandibular teeth	Gates-Glidden and K files	1.3	Calcium hydroxide paste	Provisional sealing. Not reported	After instrumentation	Visual analog scale	1st, 2nd and 3rd day	55 (using articulating paper)	55 (occlusal surface reduced by 1 mm)	No statistical significance
Creech et al. (1984)^10^	Not reported	Posterior teeth	Step-back technique	2.5	No ICM (sterile cotton pellet)	Provisional sealing: Cavit (based on zinc oxide)	After instrumentation	Questionnaire	4, 8, 24, 36 and 48 h	25 (using articulating paper)	24 (occlusal surface reduced by 0.5 mm)	No statistical significance
Emara et al. (2019)^11^	Symptomatic irreversible pulpitis; symptomatic apical periodontitis	Mandibular posterior teeth	Crown-down technique Manual files and Revo-S rotary system	2.5	No ICM (sterile cotton pellet)	After instrumentation: MD-Temp (based on zinc oxide)	After instrumentation	Visual analog scale	After instrumentation: 6, 12, 24 and 48 h	22 (using articulating paper)	22 (not report the extent of occlusal reduction)	Significant results of reduction in postoperative pain levels 12 h after instrumentation and obturation
						After obturation: Resin and/or ceramic crown	After obturation		After obturation: 6 and 12 h			
Parirokh et al. (2013)^5^	Symptomatic irreversible pulpitis	Premolar and molars	Manual files, Gates-Glidden drills and HERO 642 rotary instruments	1.3	Calcium hydroxide paste	Provisional sealing: Cotosol (based on zinc oxide)	After instrumentation	Visual analog scale	6, 12, 18, 24 h , 2nd, 3rd, 4th, 5th and 6th day	21 (using articulating paper)	25 (occlusal surface reduced by 1 mm)	No statistical significance
Raza et al. (2016)^23^	Symptomatic irreversible pulpitis	posterior maxillary and mandibular teeth	Gates-Glidden and K files	1.3	Calcium hydroxide paste	Provisional sealing. Not reported	After instrumentation	Visual analog scale	24 h	55 (using articulating paper)	55 (occlusal surface reduced by 1 mm)	No statistical significance
Rosenberg et al. (1998)^13^	Without specifications	Posterior teeth	Step-back technique Manual files	2	No ICM (sterile cotton pellet)	Provisional sealing: Cavit (based on zinc oxide)	After instrumentation	Questionnaire	Over 48 h	Not reported (using articulating paper)	Not reported (occlusal surface reduced by 0.5 to 1.0 mm)	Occlusal reduction aids in the prevention of postoperative pain in teeth with vital pulp, percussion sensitivity, preoperative pain, and/or absence of periradicular radiolucency
Sheikh et al. (2015)^15^	Symptomatic irreversible pulpitis	Posterior teeth	Manual instruments, Gates-Glidden drills and ProTaper F1 or F2 rotary instruments	3	Calcium hydroxide paste	Provisional sealing: Cavit (based on zinc oxide)	After instrumentation	Visual analog scale	6, 12, 18, 24 h, 2nd, 3rd, 4th, 5th and 6th day	201 (using articulating paper)	201 (occlusal surface reduced by 1 mm)	The mean pain score was significant 6 days after instrumentation
Viana et al. (2020)^24^	Symptomatic irreversible pulpitis	Maxillary and mandibular molars	Proglider and WaveOne Gold systems	2.5	–	Provisional sealing: Glass ionomer	After obturation	Verbal rating scale and numerical rating scale	6, 24 and 72 h	40 (using articulating paper)	38 (not report the extent of occlusal reduction)	No statistical significance
Zaman and Ahmed (2016)^22^	Symptomatic irreversible pulpitis	posterior maxillary and mandibular teeth	Gates-Glidden and K files	2.5	Calcium hydroxide paste	Provisional sealing: Cavit (based on zinc oxide)	After instrumentation	Visual analog scale	24 h, 2nd, 3rd, 4th, 5th and 6th day	125 (using articulating paper)	125 (occlusal surface reduced by 1 mm)	The mean pain score was significant 6 days after instrumentation
Zeidan (2016)^16^	Symptomatic irreversible pulpitis	Premolars	Manual instruments and WaveOne Primary	2	Endoseptone	Resin-reinforced glass ionomer	After instrumentation	Verbal rating scale	12, 24 and 48 h	20 (using articulating paper)	20 (occlusal surface reduced by 1 mm)	No statistical significance

Included teeth had initial diagnosis [AAE/ABE, 2013] of symptomatic irreversible pulpitis^5,9,15,16,22–24^, symptomatic apical periodontitis^8^, or both symptomatic irreversible pulpitis and symptomatic apical periodontitis^11,21^. In the remaining two studies, the initial diagnosis was not reported^10^, or the diagnostic criteria were not described for the clinical trial^13^.

All studies included posterior teeth (premolar and molars). One study^16^ only evaluated premolars, and two other studies^8,24^ only evaluated molars. Four studies^9,22–24^ mentioned including maxillary or mandibular teeth. Two studies^11,21^ evaluated only mandibular teeth.

For chemical–mechanical preparation, ten studies^5,8,9,11,15,16,21–24^ used the crown-down technique and the remaining two studies^10,13^ used the step-back technique. The instrumentation techniques also varied: rotary systems^8,11,15,21^, reciprocating systems^16,24^, manual systems^9,13,22,23^ and one study^10^ did not mention the instruments used.

The use of calcium hydroxide paste^5,9,15,22,23^ and camphor-chlorophenol-thymol paste with dexamethasone (Endoseptone)^16^ were mentioned as intracanal medication. The remaining four studies did not mention the use of any medication^10,11,13,21^.

The obturation techniques employed were matched single cones^8^, lateral condensation^8,11,21^, and thermomechanical compaction^24^. Two studies^8,24^ performed the single-session obturation and used a resin epoxy-based sealer, AH plus (Dentsply Maillefer, Switzerland). However, post-obturation pain assessments used different scales for each study. Emara et al.^11^ and Ahmed et al.^21^ performed endodontic treatment in two sessions, without intracanal medication and the used Adseal (Meta, Biomed, Cheongju, South Korea), a resin epoxy-based sealer.

The extent of the occlusal surface reduction ranged from 0.5 mm^10^, 0.5–1 mm^13^, and 1 mm^5,9,15,16,22,23^. The remaining studies did not report the extent of occlusal reduction^8,11,21,24^.

Pain assessment methods after endodontic treatment varied. Visual analog scale (VAS) was the most used method^5,8,9,11,15,21–23^. Other methods such as verbal rating scale—VRS^16,24^, numerical rating scale—NRS^24^, and a questionnaire application^10,13^ were also used.

Postoperative pain was mainly assessed after instrumentation^5,9,10,13,15,16,22,23^ Emara et al.^11^, and Ahmed et al.^21^ examined postoperative pain after instrumentation and obturation in two visits. Arslan et al.^8^ and Viana^24^ evaluated after obturation in a single visit. The period of pain assessment ranged from four hours after treatment^10^ to 7 days^8^, with most studies measuring pain after 24 and 48 h^5,9–11,15,16,21–23^.

The restorations were performed with a resin composites^8,11,16^, glass ionomer^24^ or with a provisional sealing material based on zinc oxide^5,10,11,13,15,21,22^. Emara et al.^11^ and Ahmed et al.^21^ have reported that patients were referred to the Prosthodontics Department for final tooth restoration. Emara et al.^11^ even mention that at the end of the study and full ceramic crowns were advised for the patients in the intervention group. Asghar et al.^9^ and Raza et al.^23^ did not report the sealing material used.

The results of these primary studies are reported as the number of patients who experienced pain (presence or absence)^5,9–11,15,16,21,23,24^.

### Risk of bias in individual studies

**Figure 2:**
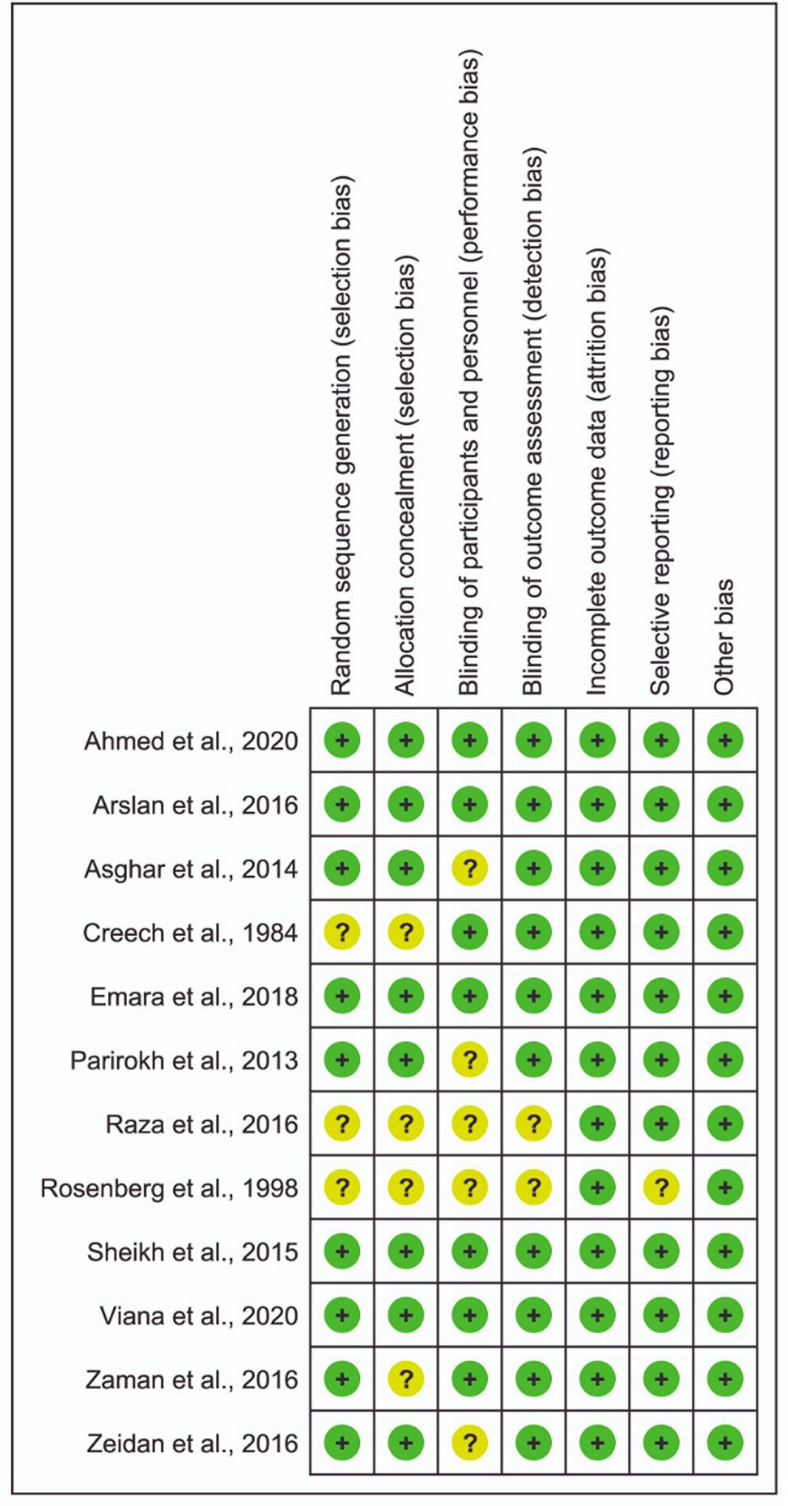
Risk of bias assessment of the included studies.

The risk of bias in each study is shown in Fig. 2. Although all included studies are considered randomized clinical trials, three studies^10,13,23^ did not explain the random sequence generation, and four studies^10,13,22,23^ did not explain the method used for allocation concealment. Five studies^5,9,13,16,23^ did not provide information about the blinding of participants.

Blinding of outcome assessment and selective outcome reporting were classified as uncertain in only two study^13^. None of the studies had attrition bias due to incomplete outcome data.

All items evaluated in five studies^8,11,15,21,24^ were classified as low risk of bias. The risk questions answered as uncertain were related to insufficient or absent information.

### Meta-analysis

Meta-analysis for post instrumentation pain was performed with eight studies^5,9–11,15,16,21,23^ using different scales. The division into subgroups was done according to the analyzed moment (post obturation and post instrumentation). During data extraction, further subgroups were considered regarding the time interval that the measurements were performed (6, 12, 24, 48 h).

Meta-analysis for post obturation of the root canal system pain was performed with three studies^11,21,24^ after 06 and 12 h using a VAS. The remaining studies differed in terms of the pain assessment scale and assessment period, which impaired the analysis. The meta-analysis was carried out using the total number of patients in each experimental group and the number of patients with pain, irrespective of its intensity. Forest plots of comparison were constructed.

Meta-analysis for post instrumentation pain did not reveal a significant difference in the reduction of postoperative pain levels after occlusal reduction after 6 h (p = 0.71; RR: 0.96; 95% CI 0.77–1.19; I^2^ = 50%; p = 0.13), 12 h (p = 0.09; RR: 0.86; 95% CI 0.72–1.02; I^2^ = 11%; p = 0.34), 24 h (p = 0.39; RR: 0.91; 95% CI 0.73–1.13; I^2^ = 30%; p = 0.20) and 48 h (p = 0.57; RR: 0.94; 95% CI 0.74–1.18; I^2^ = 0%; p = 0.98). Meta-analysis for post obturation of the root canal system, did not reveal a significant difference in the reduction of postoperative pain levels after occlusal reduction after 6 h (p = 0.32; RR: 0.89; 95% CI 0.72–1.11; I^2^ = 0%; p = 0.49) and after 12 h (p = 0.33; RR: 0.80; 95% CI 0.51–1.25; I^2^ = 53%; p = 0.12).

### Certainty of evidence

The quality of evidence and the strength of recommendation of the main outcomes evaluated by the GRADE tool were rated as moderate. According to GRADE's definition of moderate, “The true effect is likely to be close to the estimate of the effect, but there is a possibility that it is substantially different”. However, due to categorization, some degree of arbitrariness can be inputted. There is a possibility that the true effect and the effect's estimate are substantially different (Table 3).

**Table 3:** Grade of Recommendation, Assessment, Development, and Evaluation (GRADE) based on the characteristics of studies included in the systematic review and meta-analysis.

Certainty assessment							No. of patients		Effect		Result	Certainty
No. of studies	Study design	Risk of bias	Inconsistency	Indirectness	Imprecision	Other considerations	Occlusal reduction	Occlusal adjustment	Relative (95% CI)	Absolute (95% CI)	Risk ratio M–H, random, 95% CI	
3	Randomised trials	Not serious	Not serious	Not serious	Serious^a^	None	149/201 (74.1%)	149/197 (75.6%)	RR 0.96 (0.77–1.19)	30 fewer per 1000 (from 174 fewer to 144 more)	0.96 [0.77, 1.19]	⊕⊕⊕◯ MODERATE
4	Randomised trials	Not serious	Not serious	Not serious	Serious^a^	None	131/241 (54.4%)	148/237 (62.4%)	RR 0.86 (0.72–1.02)	87 fewer per 1000 (from 175 fewer to 12 more)	0.86 [0.72, 1.02]	⊕⊕⊕◯ MODERATE
7	Randomised trials	Not serious	Not serious	Not serious	Serious^a^	NONE	147/370 (39.7%)	173/367 (47.1%)	RR 0.91 (0.73–1.13)	42 fewer per 1000 (from 127 fewer to 61 more)	0.91 [0.73, 1.13]	⊕⊕⊕◯ MODERATE
6	Randomised trials	Not serious	Not serious	Not serious	Serious^a^	None	95/320 (29.7%)	101/317 (31.9%)	RR 0.94 (0.74 to 1.18)	19 fewer per 1000 (from 83 fewer to 57 more)	0.94 [0.74, 1.18]	⊕⊕⊕◯ MODERATE
3	Randomised trials	Not serious	Not serious	Not serious	Serious^a^	None	83/214 (38.8%)	94/216 (43.5%)	RR 0.89 (0.72 to 1.11)	48 fewer per 1000 (from 122 fewer to 48 more)	0.89 [0.72, 1.11]	⊕⊕⊕◯ MODERATE
3	Randomised trials	Not serious	Not serious	Not serious	Serious^a^	None	62/214 (29.0%)	75/216 (34.7%)	RR 0.80 (0.51 to 1.25)	69 fewer per 1000 (from 170 fewer to 87 more)	0.80 [0.51, 1.25]	⊕⊕⊕◯ MODERATE

GRADE approach results in an assessment of the quality of a body of evidence High: Very confident that the true effect lies close to that of the estimate of the effect.

Moderate: Moderately confident in the effect estimate, the true effect is likely to be close to the estimate of the effect, but there is a possibility that it is substantially different. Low: Limited confidence in the effect estimate, the true effect may be substantially different from the estimate of the effect.

Very low: Little confidence in the effect estimate, the true effect is likely to be substantially different from the estimate of effect.

^a^The confidence interval (CI) cross the clinical decision threshold between recommending and not recommending treatment.

## Discussion

The results of this systematic review show that occlusal reduction does not interfere with pain after the endodontic treatment in the first 48 h. Postoperative pain after endodontic treatment was separately evaluated after instrumentation and after obturation. The hypothesis that occlusal reduction interferes with pain after endodontic instrumentation and obturation was rejected. The meta-analysis did not favor either studied clinical approach. This systematic review included only clinical trials. However, many factors can influence the incidence of postoperative pain: the diagnosis and classification of the included teeth, the instrumentation techniques, the determination of working length, the type of irrigating solution, the use of intracanal medication, and obturation techniques.

Overall, five studies^11,13,15,21,22^ demonstrated significant results of occlusal reduction in preventing postoperative pain after instrumentation. However, in three of these five studies, the experimental period was the sum of pain incidence over 48 h^13^ or up to 6 days^15,22^. Postoperative pain should not be summed over experimental time points since it does not correspond to the incidence during a given period. In this respect, only Emara et al.^11^ and Ahmed et al.^21^ were included in the meta-analysis. They demonstrated a decrease in postoperative pain 12 h after occlusal reduction for both chemical–mechanical preparation and obturation.

The presence of preoperative pain can also influence the presence of postoperative pain^25,26^. Most of the meta-analysis studies^9,11,15,16,21–24^ specified pulp vitality as teeth with vital pulp, percussion sensitivity, and preoperative pain. Postoperative pain is significantly associated with previous painful symptoms in teeth without periradicular lesions, probably due to the lack of space for pressure release during instrumentation^27^ However, Alí et al.^26^ reported that pulp vitality does not affect postoperative pain intensity or frequency. Only two studies^8,11^ emphasized the periapical diagnosis, including teeth with symptomatic apical periodontitis. Therefore, symptomatic irreversible pulpitis diagnosis may not interfere with pain after endodontic treatment with occlusal reduction.

A higher incidence of pain after endodontic treatment has been reported in molars. A higher number of canals can favor periapical pain^25,28^. In this review, most of the evaluated studies^21–23^ included molars and premolars, favoring the occurrence of postoperative pain in control groups (maintenance of normal occlusal contacts) and less pain in the intervention group (occlusal reduction). However, this meta-analyzes showed no significant differences.

The instrumentation techniques used were rotary systems in five studies^5,8,11,15,21^ reciprocating systems in two^16,24^, and manual systems in five other studies^9,10,13,22,23^ Instrumentation techniques as the modified step-back, reciprocating, and rotary systems have been shown to cause postoperative pain^29^. Mechanical instruments such as continuous rotary and reciprocating systems are equivalent in terms of postoperative pain^30,31^. Few studies in the literature compare manual preparation techniques and automated systems^32^. Previous systematic reviews showed that rotary instruments' use contributed to a lower incidence and intensity of postoperative pain than manual files after single-visit root canal treatment. The use of multiple rotary-file systems contributed to a lower incidence of postoperative pain than reciprocating systems^32^. In the present review, three studies^11,15,21^ that performed rotary systems significantly reduced postoperative pain after occlusal reduction, although metanalysis did not reveal significant differences.

Concerning the irrigating solution, sodium hypochlorite (1.25–3%) was the irrigant solution in all selected studies. There is no consensus on the optimal concentration of sodium hypochlorite for root canal preparation^33^. Higher concentrations of sodium hypochlorite are more cytotoxic but have greater tissue dissolution capacity^34^. However, solutions of 5.25% sodium hypochlorite have been associated with lower postoperative pain. In the first 72 h, lower postoperative pain was observed after single-visit root canal treatment compared to 2.5% sodium hypochlorite in teeth with irreversible pulpitis^33^ and 1.3% sodium hypochlorite in necrotic pulps^35^. Studies^11,15,16,21^ that used concentration of sodium hypochlorite (2.5%) exhibited a significant reduction in postoperative pain after occlusal reduction. The sodium hypochlorite concentration used in the selected studies did not influence pain after endodontic treatment with occlusal reduction.

The determination of working length was mostly done using an apex locator followed by periapical radiography^5,9,11,15,21,23,24^. Tuncer and Gerek^36^ revealed no difference in postoperative pain between working length measurement with electronic apex locator and digital radiography. Furthermore, Arslan et al.^8^ showed that simultaneous working length measurement and root canal preparation reduce postoperative pain, causing less damage to periapical tissues. The working length varied: in the foramen^24^, at 0.5 mm from the apex^11,21^, and 1 mm from the apex^5,15,16^. Studies with working length at 0.5 mm^11,21^ from the apex and 1 mm^5,15,16^ from the apex demonstrated significant results of lower postoperative pain after occlusal reduction.

The type of restorations after endodontic treatment varied: hygroscopic materials (Cotosol, Cavit, MD-Temp)^5,10,11,13,15,21,22^, glass ionomer^24^, resin composite^8,11,16^ and ceramic crowns^11^. There are no clinical studies of postoperative pain correlating with the type of temporary restorative material used during endodontic treatment sessions.

As to the obturation, Ezpeleta et al.^37^ emphasized that postoperative pain is significantly associated with the obturation technique used during root canal treatment. However, no clinical studies correlate postoperative pain with the active obturation technique used in the included studies: lateral condensation techniques^11,21^ and thermocompaction^24^. Epoxy resin-based cement was mostly used for obturation: AHplus^8,24^ and Adseal^11,21^. No clinical studies on the incidence of postoperative pain with these endodontic cements were found. Furthermore, only two intracanal medications were used: calcium hydroxide pastes^5,9,15^, and endoseptone^16^. Four studies^10,11,16,21^ did not use any medication.

It is essential to highlight that the studies did not clarify how they measured the occlusal surface reduction of 0.5–1 mm, only mentioning the use of carbon paper and high-speed diamond tips. However, Arslan et al.^8^ used a computerized analysis system to evaluate the relative occlusal force and occlusal surface reduction. Accordingly, studies^38,39^ showed that subjective interpretation of articulating paper markings is wildly inaccurate and an ineffective clinical method for determining the relative occlusal force of tooth contacts. Sutter^39^ recommends using T-Scan, an objective method for occlusal analysis that measures the occlusal force's location.

Reducing the occlusal surface of a tooth that has the structure to be restored is an irreversible step. Also, the occlusal reduction can have a pathological repercussion for the stomatognathic system^40,41^. The present review highlights that reducing the occlusal surface does not influence postoperative pain, with moderate quality of evidence. In this sense, with endodontic treatment performed, even partially, there will be a reduction in postoperative pain. Because of the many methodological differences that can influence postoperative pain, only a small number of studies were included. However, most of these studies had a low risk of bias (“Supplementary materials”).

Among the limitations of the present study, the following stand out: language restrictions, the small number of articles included, methodological heterogeneity, the adoption of multiple pain scales, and the different follow-up periods of observation. Future studies should include investigation of occlusal surface reduction before root canal treatment to prevent postoperative pain on teeth with necrotic pulp and apical periodontitis. It is known that periapical lesions represent an increased risk of postoperative pain^27^. Randomized clinical trials on this subject are scarce so far, making it challenging to consolidate clinical protocols that preserve dental structures.

## Conclusion

The occlusal reduction does not interfere with pain after endodontic instrumentation (at 6, 12, 24, or 48 h) and the obturation (at 6 and 12 h). The certainty of evidence within the studies included in this meta-analysis was considered moderate.

## Supplementary Information

Supplementary Information.
