# Supplementary material for: Influence of occlusal reduction on pain after endodontic treatment: a systematic review and meta-analysis
Source: Sci Rep. 2021 Jul 7;11:14019. doi: 10.1038/s41598-021-93119-6 (PMC8263790; doi:10.1038/s41598-021-93119-6)
Supplement: Supplementary file 1 — Supplementary Information. [file 41598_2021_93119_MOESM1_ESM.docx]

APPENDIX (SUPPLEMENTARY MATERIAL)


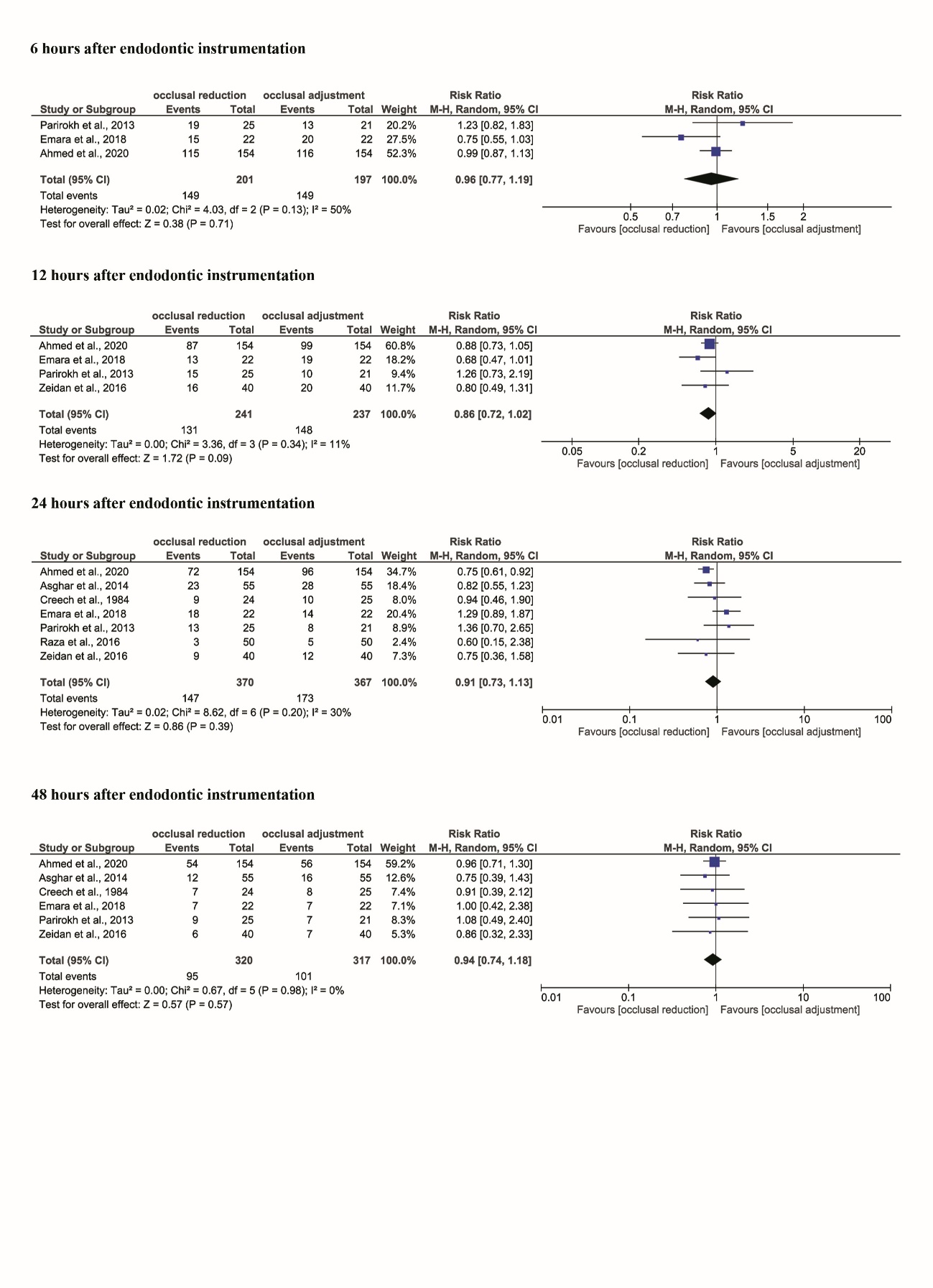


**Image 1.** Forest plots of comparison were constructed for 6, 12, 24 and 48 hours after endodontic instrumentation.

**
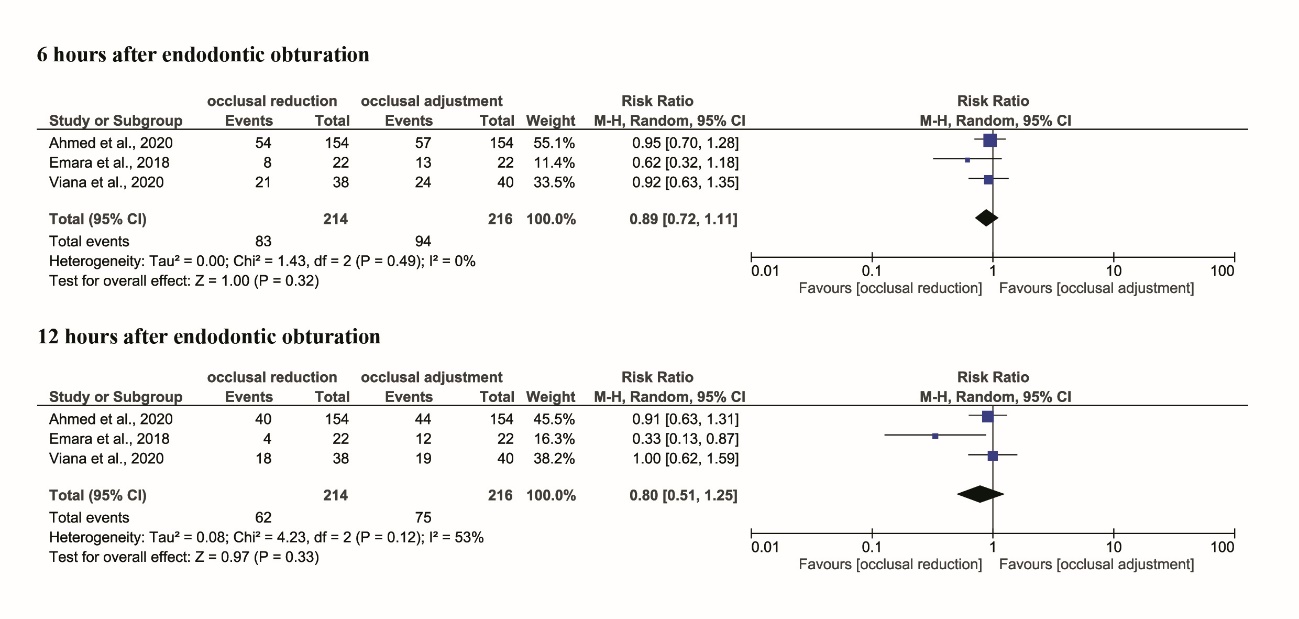
**

**Image 2.** Forest plots of comparison were constructed for 6 and 12 hours after endodontic obturation.
